# Tobacco Product Use Among Middle and High School Students — United States, 2022

**DOI:** 10.15585/mmwr.mm7145a1

**Published:** 2022-11-11

**Authors:** Eunice Park-Lee, Chunfeng Ren, Maria Cooper, Monica Cornelius, Ahmed Jamal, Karen A. Cullen

**Affiliations:** ^1^Center for Tobacco Products, Food and Drug Administration, Silver Spring, Maryland; ^2^Office on Smoking and Health, National Center for Chronic Disease Prevention and Health Promotion, CDC.

Tobacco use* is the leading cause of preventable disease, disability, and death among adults in the United States (*1*). Youth use of tobacco products in any form is unsafe, and nearly all tobacco use begins during youth and young adulthood (*2*). The Food and Drug Administration (FDA) and CDC analyzed data from the 2022 National Youth Tobacco Survey (NYTS) to estimate current (past 30-day) use of eight tobacco products among U.S. middle (grades 6–8) and high school (grades 9–12) students. In 2022, approximately 11.3% of all students (representing 3.08 million persons) reported currently using any tobacco product, including 16.5% of high school and 4.5% of middle school students (2.51 million and 530,000 persons, respectively). Electronic cigarettes (e-cigarettes) were the most commonly used tobacco product among high school (14.1%; 2.14 million) and middle school (3.3%; 380,000) students. Approximately 3.7% of all students (representing 1 million persons) reported currently smoking any combustible tobacco product. Current use of any tobacco product was higher among certain population groups, including 13.5% of non-Hispanic American Indian or Alaska Native (AI/AN)^†^ students; 16.0% of students identifying as lesbian, gay, or bisexual (LGB); 16.6% of students identifying as transgender; 18.3% of students reporting severe psychological distress; 12.5% of students with low family affluence; and 27.2% of students with low academic achievement. Implementation of comprehensive evidence-based tobacco control strategies, combined with FDA regulation, is important for preventing and reducing youth tobacco product use (*1*,*2*).

NYTS is a cross-sectional, voluntary, school-based, self-administered survey of U.S. middle and high school students. A stratified, three-stage cluster sampling procedure generated a nationally representative sample of U.S. students attending public and private schools in grades 6–12. In 2022, the survey was administered during January 18–May 31, 2022. The 2022 NYTS was conducted using an online survey, with nearly all (99.3%) students completing it on a school campus. In total, 28,291 students (student participation rate = 76.1%) from 341 schools (school participation rate = 59.4%) participated, yielding an overall response rate of 45.2%. Because of changes in methodology, including differences in survey administration and data collection procedures, the ability to compare estimates from 2022 with those from previous NYTS waves is limited; differences between estimates might result from changes in methodology, actual behavior, or both.^§^ Detailed information about NYTS is available online.^¶^

Weighted prevalence estimates and population totals** of current use of eight tobacco products (e-cigarettes, cigars, cigarettes, smokeless tobacco,^††^ hookahs, nicotine pouches,^§§^ heated tobacco products, and pipe tobacco) and three composite measures (use of any tobacco product,^¶¶^ any combustible tobacco product,*** and multiple tobacco products^†††^) were reported among all students and by school level; current use was defined as use on ≥1 day during the past 30 days. In addition, estimates of current use of any tobacco product were reported by selected demographic characteristics^§§§^ and indicators of social determinants of health.^¶¶¶^ Estimates with a relative SE of >30% or an unweighted denominator of <50 were suppressed. Analyses were conducted using SAS-callable SUDAAN (version 11.0.4; RTI International). The 2022 NYTS was approved by the Office of Management and Budget, the contracted data collectors’ institutional review board (IRB), and CDC’s IRB.****

In 2022, among middle and high school students, 24.8% reported ever having used any tobacco product (Supplementary Table, https://stacks.cdc.gov/view/cdc/122048), and 11.3% reported current use of any tobacco product (Table 1). Overall, current use of any tobacco product was reported by 12.3% of female, 10.3% of male, 13.5% of AI/AN, 13.0% of non-Hispanic multiracial (multiracial), 12.4% of non-Hispanic White (White), 11.5% of non-Hispanic Black or African American (Black), 11.1% of Hispanic or Latino (Hispanic), and 3.1% of non-Hispanic Asian (Asian) students. Current use of any combustible tobacco product was reported by 5.7% of Black, 4.7% of multiracial, 4.6% of AI/AN, 3.9% of Hispanic, and 3.4% of White students. Notably, current use of any combustible tobacco product was highest among Black students, specifically for cigar (3.3%) and hookah (2.3%) use. Estimates among non-Hispanic Native Hawaiian or other Pacific Islander (NH/OPI) students, overall and by school level, were statistically unreliable for all current use measures and are not reported.

**TABLE 1:** Percentage of middle and high school students who reported current (past 30-day) tobacco product use, by product,* overall and by school level, sex, and race and ethnicity — National Youth Tobacco Survey, United States, 2022

Tobacco product	Sex, % (95% CI)		Race and ethnicity,^†^ % (95% CI)						Total	
	Female	Male	White, NH	Black or African American, NH	Hispanic	Asian, NH	AI/AN, NH	Multiracial, NH	% (95% CI)	Estimated no. of users^§^
**Overall**										
Any tobacco product^¶^	12.3 (10.7–14.1)	10.3 (8.6–12.4)	12.4 (10.2–14.8)	11.5 (9.2–14.3)	11.1 (9.7–12.8)	3.1 (1.9–4.9)	13.5 (9.9–18.2)	13.0 (10.3–16.2)	**11.3 (9.7–13.1)**	**3,080,000**
E-cigarettes	10.5 (8.9–12.3)	8.3 (6.7–10.3)	11.0 (8.9–13.5)	8.2 (6.3–10.6)	8.8 (7.6–10.1)	—**	9.6 (6.6–13.8)	10.6 (8.3–13.5)	**9.4 (8.0–11.1)**	**2,550,000**
Cigars	1.5 (1.1–1.9)	2.2 (1.6–3.0)	1.8 (1.3–2.5)	3.3 (2.3–4.7)	1.7 (1.3–2.3)	—	—	2.2 (1.3–3.7)	**1.9 (1.4–2.4)**	**500,000**
Cigarettes	1.5 (1.2–2.0)	1.7 (1.3–2.2)	1.8 (1.4–2.3)	—	1.8 (1.3–2.5)	—	—	2.3 (1.4–3.9)	**1.6 (1.3–2.0)**	**440,000**
Smokeless tobacco (composite)^††^	0.8 (0.6–1.1)	1.7 (1.3–2.2)	1.5 (1.2–2.0)	—	1.2 (0.8–1.7)	—	—	—	**1.3 (1.0–1.6)**	**330,000**
Hookahs	1.1 (0.8–1.4)	1.1 (0.9–1.5)	0.7 (0.5–1.0)	2.3 (1.7–3.1)	1.5 (1.1–2.1)	—	—	1.0 (0.6–1.6)	**1.1 (0.9–1.4)**	**290,000**
Nicotine pouches	0.7 (0.5–1.0)	1.4 (1.0–2.0)	1.3 (0.9–1.9)	—	1.0 (0.7–1.5)	—	—	—	**1.1 (0.8–1.4)**	**280,000**
Heated tobacco products	0.9 (0.7–1.2)	1.0 (0.8–1.5)	0.8 (0.6–1.0)	0.8 (0.5–1.3)	1.4 (0.9–2.0)	—	—	—	**1.0 (0.8–1.2)**	**260,000**
Pipe tobacco	0.5 (0.3–0.7)	0.7 (0.5–1.0)	0.5 (0.3–0.7)	—	0.8 (0.5–1.3)	—	—	—	**0.6 (0.4–0.8)**	**150,000**
Any combustible tobacco product^§§^	3.5 (2.9–4.3)	3.9 (3.1–4.8)	3.4 (2.7–4.2)	5.7 (4.3–7.5)	3.9 (3.1–4.9)	—	4.6 (2.8–7.5)	4.7 (3.3–6.6)	**3.7 (3.1–4.5)**	**1,000,000**
Multiple tobacco products^¶¶^	3.1 (2.5–3.8)	3.9 (3.1–5.0)	3.8 (3.0–4.9)	3.7 (2.7–5.2)	3.5 (2.7–4.5)	—	3.5 (2.2–5.5)	3.9 (2.7–5.6)	**3.5 (2.9–4.4)**	**960,000**
**High school (grades 9–12)**										
Any tobacco product^¶^	17.6 (15.8–19.6)	15.3 (13.0–17.9)	18.6 (16.2–21.2)	15.2 (12.0–19.2)	14.9 (13.0–17.1)	—	20.0 (13.9–28.1)	17.8 (14.0–22.4)	**16.5 (14.6–18.5)**	**2,510,000**
E-cigarettes	15.4 (13.6–17.4)	12.8 (10.6–15.3)	16.9 (14.6–19.5)	11.1 (8.3–14.7)	12.2 (10.7–14.0)	—	14.6 (9.4–22.0)	14.3 (11.0–18.3)	**14.1 (12.4–16.0)**	**2,140,000**
Cigars	2.1 (1.5–2.8)	3.5 (2.6–4.6)	2.8 (2.1–3.8)	4.4 (2.9–6.8)	2.2 (1.7–2.9)	—	—	3.4 (2.0–5.7)	**2.8 (2.2–3.5)**	**410,000**
Cigarettes	1.8 (1.4–2.3)	2.3 (1.8–3.0)	2.4 (1.8–3.0)	—	2.0 (1.3–3.1)	—	—	3.6 (2.0–6.1)	**2.0 (1.7–2.5)**	**310,000**
Smokeless tobacco (composite)^††^	0.9 (0.6–1.3)	2.3 (1.7–3.1)	2.2 (1.7–3.0)	—	1.3 (0.9–1.9)	—	—	—	**1.6 (1.3–2.1)**	**240,000**
Hookahs	1.3 (1.0–1.9)	1.7 (1.3–2.3)	1.1 (0.8–1.6)	3.4 (2.5–4.5)	1.7 (1.2–2.5)	—	—	—	**1.5 (1.2–1.9)**	**220,000**
Nicotine pouches	0.8 (0.5–1.2)	2.1 (1.5–2.9)	2.0 (1.4–2.9)	—	1.1 (0.7–1.8)	—	—	—	**1.4 (1.1–2.0)**	**210,000**
Heated tobacco products	1.0 (0.7–1.4)	1.3 (0.9–1.8)	1.1 (0.8–1.5)	—	1.4 (0.8–2.4)	—	—	—	**1.1 (0.9–1.5)**	**160,000**
Pipe tobacco	0.5 (0.3–0.8)	0.9 (0.6–1.3)	0.6 (0.4–1.1)	—	0.8 (0.5–1.3)	—	—	—	**0.7 (0.5–1.0)**	**100,000**
Any combustible tobacco product^§§^	4.7 (3.8–5.7)	5.8 (4.8–7.0)	4.9 (3.9–6.0)	8.2 (6.1–10.9)	4.9 (3.8–6.2)	—	6.6 (3.7–11.5)	7.4 (5.4–10.1)	**5.2 (4.4–6.2)**	**790,000**
Multiple tobacco products^¶¶^	4.1 (3.4–5.0)	5.9 (4.6–7.4)	5.7 (4.5–7.1)	5.5 (3.8–7.8)	4.1 (3.1–5.4)	—	4.6 (2.6–8.1)	5.6 (3.7–8.3)	**5.0 (4.1–6.1)**	**760,000**
**Middle school (grades 6–8)**										
Any tobacco product^¶^	5.3 (4.0–7.0)	3.8 (3.1–4.6)	3.7 (2.6–5.1)	6.2 (4.7–8.2)	5.7 (4.6–7.0)	1.3 (0.8–2.2)	6.2 (3.6–10.4)	6.8 (4.0–11.3)	**4.5 (3.7–5.5)**	**530,000**
E-cigarettes	4.1 (2.9–5.7)	2.5 (2.0–3.3)	2.8 (1.9–4.3)	4.1 (2.7–6.1)	4.2 (3.2–5.4)	—	—	6.0 (3.3–10.5)	**3.3 (2.6–4.2)**	**380,000**
Cigars	0.6 (0.4–1.0)	0.6 (0.4–0.9)	—	1.7 (1.0–2.7)	0.8 (0.5–1.4)	—	—	—	**0.6 (0.4–0.9)**	**70,000**
Cigarettes	1.1 (0.6–2.0)	0.8 (0.5–1.3)	—	—	1.2 (0.8–1.9)	—	—	—	**1.0 (0.6–1.5)**	**110,000**
Smokeless tobacco (composite)^††^	0.6 (0.4–0.9)	0.7 (0.5–1.1)	0.6 (0.3–0.9)	—	—	—	—	—	**0.7 (0.5–1.0)**	**80,000**
Hookahs	0.6 (0.4–0.8)	0.4 (0.2–0.6)	—	—	0.9 (0.6–1.4)	—	—	—	**0.5 (0.4–0.7)**	**50,000**
Nicotine pouches	—	0.4 (0.2–0.7)	—	—	0.6 (0.4–1.0)	—	—	—	**0.5 (0.3–0.8)**	**50,000**
Heated tobacco products	0.7 (0.4–1.3)	0.6 (0.4–0.9)	0.4 (0.2–0.7)	—	0.9 (0.5–1.5)	—	—	—	**0.7 (0.5–0.9)**	**70,000**
Pipe tobacco	—	0.3 (0.2–0.6)	—	—	—	—	—	—	**0.3 (0.2–0.4)**	**30,000**
Any combustible tobacco product^§§^	1.9 (1.2–2.8)	1.4 (1.0–1.9)	1.3 (0.7–2.3)	2.2 (1.4–3.3)	2.2 (1.6–3.0)	—	—	—	**1.6 (1.2–2.2)**	**190,000**
Multiple tobacco products^¶¶^	1.7 (1.1–2.7)	1.3 (0.9–1.7)	1.3 (0.7–2.3)	1.3 (0.8–2.3)	2.1 (1.5–2.9)	—	—	—	**1.5 (1.1–2.1)**	**170,000**

**Abbreviations:** AI/AN = American Indian or Alaska Native; NH = non-Hispanic; NH/OPI = Native Hawaiian or other Pacific Islander.

* Current use is defined as use on ≥1 day during the past 30 days for each product. Because of missing data on past 30-day use questions, denominators for each tobacco product might differ.

^†^ The race and ethnicity measure used in analyses allowed for multiple races, which differs from the measure used in previous NYTS publications that categorized all respondents into single race and ethnicity groups. Hispanic persons could be of any race (White, Black or African American, Asian, AI/AN, NH/OPI, or multiracial). Estimates among NH NH/OPI students, overall and by school level, were statistically unreliable for all measures and are omitted.

^§^ Estimated weighted total number of current tobacco users was rounded down to the nearest 10,000 persons. Overall estimates were reported among 28,291 U.S. middle and high school students. School level was determined by self-reported grade level: high school (grades 9–12; n = 16,118) and middle school (grades 6–8; n = 12,041). Overall population estimates might not sum to corresponding subgroup population estimates because of rounding or inclusion of students who did not self-report sex, race and ethnicity, or grade level.

^¶^ Any tobacco product use is defined as current use of one or more of the following tobacco products on ≥1 day during the past 30 days: e-cigarettes, cigars, cigarettes, smokeless tobacco (chewing tobacco, snuff, and dip; snus; and dissolvable tobacco products), hookahs, nicotine pouches, heated tobacco products, pipe tobacco, or bidis (small brown cigarettes wrapped in a leaf).

** Dashes indicate that data were statistically unreliable because of an unweighted denominator <50 or a relative SE >30%.

^††^ Smokeless tobacco was defined as chewing tobacco, snuff, and dip; snus; and dissolvable tobacco products.

^§§^ Any combustible tobacco product use was defined as current use of one or more of the following tobacco products on ≥1 day during the past 30 days: cigars, cigarettes, hookahs, pipe tobacco, or bidis.

^¶¶^ Multiple tobacco product use was defined as current use of two or more of the following tobacco products on ≥1 day during the past 30 days: e-cigarettes, cigars, cigarettes, smokeless tobacco, hookahs, nicotine pouches, heated tobacco products, pipe tobacco, or bidis.

Tobacco use prevalence varied by certain demographic characteristics and indicators of social determinants of health. By sexual identity, current use of any tobacco product was 16.0% among middle and high school students identifying as LGB, 9.7% among those identifying as heterosexual, and 7.1% among those who were not sure. In addition, current use of any tobacco product was reported by 16.6% of students identifying as transgender, 14.5% of those who were not sure, and 10.2% of those identifying as not transgender (Table 2). Prevalence of current tobacco product use ranged from 7.2% among students reporting no psychological distress to 18.3% among those reporting severe distress.^††††^ Current use of any tobacco product was reported by 12.5% of students whose families were categorized as having low affluence,^§§§§^ and by 9.6% of those with medium or high family affluence. Current tobacco product use was inversely related to self-reported grades in school, ranging from 6.6% among those reporting “mostly As” to 27.2% among those reporting “mostly Fs.” Current tobacco product use was higher among students who spoke English in the home (11.1%) compared with those who spoke another language at home (8.5%).

**TABLE 2:** Percentage of middle and high school students who reported current (past 30-day) use of any tobacco product,* overall^†^ and by school level, selected demographic characteristics, and indicators of social determinants of health — National Youth Tobacco Survey, United States, 2022

Characteristic	Overall		High school (grades 9–12)		Middle school (grades 6–8)	
	% (95% CI)	Estimated no. of users^§^	% (95% CI)	Estimated no. of users^§^	% (95% CI)	Estimated no. of users^§^
**Sexual identity**						
Heterosexual	**9.7 (8.2–11.5)**	**1,750,000**	14.1 (12.2–16.2)	1,490,000	3.6 (2.9–4.4)	260,000
Gay, lesbian, or bisexual	**16.0 (13.8–18.4)**	**630,000**	21.5 (18.7–24.5)	500,000	7.6 (5.5–10.3)	120,000
Not sure	**7.1 (5.1–9.8)**	**170,000**	12.5 (9.1–16.9)	120,000	—^¶^	—
**Transgender**						
No, not transgender	**10.2 (8.6–12.0)**	**2,200,000**	14.8 (12.8–16.9)	1,860,000	3.8 (3.1–4.7)	340,000
Yes, transgender	**16.6 (13.0–21.0)**	**130,000**	20.5 (15.2–27.0)	90,000	9.1 (6.0–13.6)	30,000
Not sure	**14.5 (10.7–19.3)**	**120,000**	23.6 (16.7–32.4)	90,000	—	—
I don’t know what this question is asking	**8.1 (5.9–10.9)**	**80,000**	13.8 (10.0–18.7)	50,000	4.0 (2.3–6.8)	20,000
Psychological distress**						
None	**7.2 (6.0–8.5)**	**900,000**	11.0 (9.5–12.8)	760,000	2.3 (1.8–3.0)	120,000
Mild	**10.9 (8.9–13.3)**	**510,000**	16.4 (13.9–19.3)	460,000	2.5 (1.7–3.8)	40,000
Moderate	**13.1 (10.4–16.3)**	**400,000**	18.1 (15.0–21.7)	340,000	5.3 (2.9–9.4)	60,000
Severe	**18.3 (15.7–21.4)**	**540,000**	23.5 (20.6–26.6)	410,000	10.6 (8.0–13.8)	120,000
**Family Affluence Scale^††^**						
Low	**12.5 (11.2–13.9)**	**680,000**	17.2 (15.5–19.0)	570,000	5.1 (4.0–6.5)	100,000
Medium	**9.6 (7.8–11.8)**	**870,000**	13.8 (11.3–16.7)	720,000	4.0 (3.3–4.9)	150,000
High	**9.6 (7.8–11.8)**	**900,000**	15.1 (12.9–17.6)	770,000	2.8 (2.0–4.0)	120,000
**Grades in school**						
Mostly As	**6.6 (5.2–8.2)**	**710,000**	10.1 (8.4–12.2)	580,000	2.4 (1.7–3.3)	110,000
Mostly Bs	**11.3 (9.4–13.6)**	**850,000**	16.4 (14.0–19.2)	750,000	3.3 (2.6–4.3)	90,000
Mostly Cs	**16.5 (13.8–19.6)**	**490,000**	21.1 (17.9–24.7)	410,000	7.7 (5.6–10.4)	70,000
Mostly Ds	**22.7 (18.7–27.3)**	**170,000**	28.9 (23.8–34.7)	140,000	11.5 (7.3–17.6)	30,000
Mostly Fs	**27.2 (22.0–33.2)**	**140,000**	30.6 (23.6–38.6)	100,000	19.2 (12.3–28.8)	30,000
**Speak language other than English at home**						
Yes	**8.5 (7.1–10.3)**	**640,000**	12.1 (10.2–14.2)	460,000	4.7 (3.4–6.3)	160,000
No	**11.1 (9.4–13.0)**	**1,890,000**	16.1 (14.1–18.4)	1,630,000	3.6 (2.8–4.5)	250,000

* Any tobacco product use is defined as current use of one or more of the following tobacco products on ≥1 day during the past 30 days: e-cigarettes, cigarettes, cigars, smokeless tobacco (chewing tobacco, snuff, and dip; snus; and dissolvable tobacco products), hookahs, heated tobacco products, nicotine pouches, pipe tobacco, or bidis (small brown cigarettes wrapped in a leaf).

^†^ Overall estimates were reported among 28,291 U.S. middle and high school students. School level was determined by self-reported grade level: high school (grades 9–12; n = 16,118) and middle school (grades 6–8; n = 12,041).

^§^ Estimated weighted total numbers of current tobacco users were rounded down to the nearest 10,000 persons. Overall estimates might not sum to corresponding subgroup estimates by school level because of rounding or inclusion of students who did not self-report grade level.

^¶^ Dashes indicate that data were statistically unreliable because of an unweighted denominator <50 or a relative SE >30%.

** Psychological distress was assessed with a composite scale comprised of four questions: “During the past two weeks, how often have you been bothered by any of the following problems?” 1) “Little interest or pleasure in doing things”; 2) “Feeling down, depressed, or hopeless”; 3) “Feeling nervous, anxious, or on edge”; and 4) “Not being able to stop or control worrying.” Complete data from all four questions (n = 24,251) were summed (range = 0–12) and categorized.

^††^ Family affluence was assessed with a composite scale comprised of four questions: 1) “Does your family own a vehicle (such as a car, van, or truck)?”; 2) “Do you have your own bedroom?”; 3) “How many computers (including laptops and tablets, not including game consoles and smartphones) does your family own?”; and 4) “During the past 12 months, how many times did you travel on vacation with your family?” Complete data from all four questions (n = 24,972) were summed (range = 0–9) and categorized into approximate tertiles based on the sample’s weighted distribution of scores.

Among high school students, 16.5% reported current use of any tobacco product, 5.2% (31.5% of current users of any tobacco product) reported current use of any combustible tobacco product, and 5.0% (30.3% of any tobacco product users) reported current use of multiple tobacco products. E-cigarettes were the product type most commonly used (14.1%), followed by cigars (2.8%), cigarettes (2.0%), smokeless tobacco (1.6%), hookahs (1.5%), nicotine pouches (1.4%), heated tobacco products (1.1%), and pipe tobacco (0.7%).

Among middle school students, 4.5% reported current use of any tobacco product, 1.6% (35.6% of current users of any tobacco product) reported current use of any combustible tobacco product, and 1.5% (33.3% of any tobacco product users) reported current use of multiple tobacco products. By product type, e-cigarettes were most commonly used (3.3%), followed by cigarettes (1.0%), smokeless tobacco and heated tobacco products (both 0.7%), cigars (0.6%), hookahs and nicotine pouches (both 0.5%), and pipe tobacco (0.3%).

## Discussion

An estimated 3.08 million U.S. middle and high school students reported current use of any tobacco product in 2022, representing approximately one in six high school students and one in 22 middle school students. Among all students who currently used any tobacco product, 31.0% reported using multiple tobacco products during the past 30 days. Multiple tobacco product use among youths is particularly concerning because it is associated with nicotine dependence, which increases the likelihood of sustained tobacco use in adulthood (*1*–*3*).

Similar to the previous year (*4*), indicators of social determinants of health were assessed in relation to tobacco product use, and in 2022, estimates for Asian, AI/AN, NH/OPI, and multiracial population groups were provided for the first time, allowing measurement of disparities affecting these groups. These findings suggested disparities in current tobacco product use among U.S. youths. Whereas AI/AN students reported the highest prevalence of current use of any tobacco product, current use of any combustible tobacco product, specifically cigar and hookah use, was highest among Black students. In addition, current use of any tobacco product was higher among those students identifying as LGB or transgender, those reporting severe psychological distress, those with low family affluence, and those with low academic achievement.

Cigarette smoking among U.S. youths has been steadily declining during the past 2 decades (*1*,*2*). Although the ability to compare results between 2022 and previous survey waves is limited because of methodological changes, approximately 3.7% of middle and high school students reported current use of any combustible tobacco product in 2022. Efforts are ongoing at the national, state, and local levels, such as enforcing the federal minimum age of sale of 21 years for all tobacco product types (*5*) and restricting the sale of flavored tobacco products in some states and communities.^¶¶¶¶^ In addition, efforts have been made to raise the price of tobacco products, prohibit public indoor use of tobacco products, and warn about the dangers of tobacco product use through media campaigns and other educational interventions (*6*). Furthermore, factors related to the ongoing COVID-19 pandemic could have possibly affected youth access to tobacco products and tobacco use (*7*).

The 2022 NYTS findings suggest ongoing disparities in tobacco product use, which, to a certain extent, might be attributed to higher volume of exposure to tobacco promotion and advertising and higher tobacco retail outlet density in racial and ethnic minority communities among other systemic environmental factors (*2*). Concerted efforts by parents, schools, and those who work with youths are necessary to reduce tobacco-related disparities by helping youths recognize and avoid the dangers of tobacco use. Furthermore, combined with regulation by FDA, efforts to address social and structural determinants of health disparities are warranted for advancing tobacco-related health equity and reducing and preventing all forms of tobacco product use among U.S. youths (*1*,*2*).

The findings in this report are subject to at least five limitations. First, data were self-reported and cross-sectional in nature; they might be subject to recall and response bias, and no causal inferences can be drawn. Second, data were collected only from middle and high school students who attended public or private schools; findings might not be generalizable to youths who are home-schooled, have dropped out of school, are in detention centers, or are enrolled in alternative schools. However, data from the Current Population Survey indicate that approximately 96% of U.S. youths aged 10–17 years were enrolled in a traditional school in 2020 (*8*). Third, the student participation rate in 2022 was lower than expected. However, weighting adjustments for nonresponse were applied to produce national weighted estimates.***** Fourth, because of small sample sizes, many estimates for racial and ethnic population groups were not reliable, especially for less prevalent tobacco products and among the NH/OPI population. Finally, the current definition of AI/AN excludes students indicating both AI/AN and another race and ethnicity, further reducing the sample size and statistical power for the AI/AN group assessed in these data.

Youth use of tobacco products in any form is unsafe. In 2022, nearly one in nine U.S. middle and high school students, or approximately 3.08 million youths overall, reported current use of any tobacco product. Continued surveillance efforts of all tobacco product types, including novel products, and sustained implementation of population-based tobacco control strategies, combined with regulation by FDA, are warranted to prevent and reduce youth tobacco product use.

**Summary**

**What is already known about this topic?**

Commercial tobacco use is the leading cause of preventable disease and death in the United States. Youth use of tobacco products in any form is unsafe.

**What is added by this report?**

In 2022, nearly one in nine (11.3%) middle and high school students reported current tobacco product use, including 13.5% of non-Hispanic American Indian or Alaska Native students; 16.0% who identified as lesbian, gay, or bisexual; 16.6% who identified as transgender; 18.3% who reported severe psychological distress; 12.5% with low family affluence; and 27.2% with low academic achievement.

**What are the implications for public health?**

Continued surveillance, sustained implementation of population-based tobacco control strategies, and efforts to address disparities, combined with the Food and Drug Administration’s regulation are warranted to prevent and reduce youth tobacco use.

## Footnotes

* The term “tobacco” as used in this report refers to commercial tobacco products and not to sacred and traditional use of tobacco by some American Indian communities.

† Respondents could select one or more races: AI/AN, Asian, Black, NH/OPI, or White. Respondents who indicated Hispanic, Latino, Latina, or Spanish origin were classified as Hispanic, irrespective of their race. Non-Hispanic respondents who selected more than one race were classified as multiracial.

§ The NYTS was conducted in schools using an electronic tablet in 2019 and 2020. Because of COVID-19 concerns, the 2021 NYTS was conducted using web-based data collection, with approximately one half (50.8%) of students completing the survey in school. The 2022 NYTS was also conducted using web-based data collection, with nearly all (99.3%) students completing the survey in school.

¶ The 2022 NYTS included additional sampling to increase the sample size of AI/AN and Asian students for analyses. All respondents completing surveys were included in the final data set. https://www.cdc.gov/tobacco/data_statistics/surveys/nyts/index.htm

** Data were weighted to account for the complex survey design and to adjust for nonresponse. Population estimates of current use were rounded down to the nearest 10,000 persons.

†† Definition of smokeless tobacco includes chewing tobacco, snuff, and dip; snus; and dissolvable tobacco products. Use of individual smokeless tobacco products is not reported.

§§ Questions assessing awareness, ever use, and current use of nicotine pouches were accompanied by a brief description, “The next section is about ‘nicotine pouches’ such as Zyn, on!, or Velo. These small, flavored pouches contain nicotine that comes from tobacco. Users place them in their mouth. Nicotine pouches are different from other smokeless tobacco products such as snus, dip, or chewing tobacco, because they do not contain any tobacco leaf.”

¶¶ Any tobacco product use was defined as current use of one or more of the following tobacco products on ≥1 day during the past 30 days: e-cigarettes, cigarettes, cigars, smokeless tobacco (chewing tobacco, snuff, and dip; snus; and dissolvable tobacco products), hookahs, heated tobacco products, nicotine pouches, pipe tobacco, or bidis (small brown cigarettes wrapped in a leaf).

*** Any combustible tobacco product use is defined as current use of one or more of the following tobacco products on ≥1 day during the past 30 days: cigarettes, cigars, hookahs, pipe tobacco, or bidis.

††† Multiple tobacco product use was defined as current use of two or more of the following tobacco products on ≥1 day during the past 30 days: e-cigarettes, cigarettes, cigars, smokeless tobacco, hookahs, heated tobacco products, nicotine pouches, pipe tobacco, or bidis.

§§§ Demographic characteristics included sex (female or male), race and ethnicity (non-Hispanic White, non-Hispanic Black, Hispanic, non-Hispanic Asian, non-Hispanic AI/AN, non-Hispanic NH/OPI, and non-Hispanic multiracial [two or more races]), sexual identity (heterosexual, LGB, or not sure), and transgender status (no, yes, not sure, or don't know what this question is asking). Race and ethnicity measure used in analyses allowed for multiple races, which is different from the measure used in previous NYTS publications that categorized respondents into single race and ethnicity groups.

¶¶¶ Indicators of social determinants of health included grades in school (mostly As, Bs, Cs, Ds, or Fs), speaking a language other than English at home (yes or no), psychological distress (none, mild, moderate, or severe), and family affluence (low, medium, or high).

**** 45 C.F.R. part 46; 21 C.F.R. part 56.

†††† Psychological distress was assessed with a composite scale comprised of four questions: “During the past two weeks, how often have you been bothered by any of the following problems?” 1) “Little interest or pleasure in doing things”; 2) “Feeling down, depressed, or hopeless”; 3) “Feeling nervous, anxious, or on edge”; and 4) “Not being able to stop or control worrying.” Complete data from all four questions (n = 24,251) were summed (range = 0–12) and categorized.

§§§§ Family affluence was assessed with a composite scale comprised of four questions: 1) “Does your family own a vehicle (such as a car, van, or truck)?”; 2) “Do you have your own bedroom?”; 3) “How many computers (including laptops and tablets, not including game consoles and smartphones) does your family own?”; and 4) “During the past 12 months, how many times did you travel on vacation with your family?” Complete data from all four questions (n = 24,972) were summed (range = 0–9) and categorized into approximate tertiles based on the sample’s weighted distribution of scores.

¶¶¶¶
https://www.tobaccofreekids.org/assets/factsheets/0398.pdf

***** The actual school and student participation rates in 2022 were lower than in previous years, possibly because of lower expected response rates being used in the sampling plan as a result of the COVID-19 pandemic and school policy. The assumed student participation rate used for the sampling plan was adjusted to account both for a growing number of ineligible students and parental refusal and for the new data collection methods (i.e., 100% virtually supported fielding methodology without in-person survey administrators). https://www.cdc.gov/tobacco/data_statistics/surveys/nyts/index.htm
